# Brain Mapping of Behavioral Domains Using Multi-Scale Networks and Canonical Correlation Analysis

**DOI:** 10.3389/fnins.2022.889725

**Published:** 2022-06-21

**Authors:** Izaro Fernandez-Iriondo, Antonio Jimenez-Marin, Basilio Sierra, Naiara Aginako, Paolo Bonifazi, Jesus M. Cortes

**Affiliations:** ^1^Computer Science and Artificial Intelligence, University of the Basque Country (UPV/EHU), San Sebastian, Spain; ^2^Computational Neuroimaging Lab, BioCruces-Bizkaia Health Research Institute, Barakaldo, Spain; ^3^Doctoral Programme in Informatics Engineering, University of the Basque Country (UPV/EHU), San Sebastian, Spain; ^4^Biomedical Research Doctorate Program, University of the Basque Country (UPV/EHU), Leioa, Spain; ^5^IKERBASQUE: The Basque Foundation for Science, Bilbao, Spain; ^6^Department of Cell Biology and Histology, University of the Basque Country (UPV/EHU), Leioa, Spain

**Keywords:** brain network mapping, multi-scale networks, functional MRI, diffusion MRI, behavior, machine learning, canonical correlation analysis

## Abstract

Simultaneous mapping of multiple behavioral domains into brain networks remains a major challenge. Here, we shed some light on this problem by employing a combination of machine learning, structural and functional brain networks at different spatial resolutions (also known as scales), together with performance scores across multiple neurobehavioral domains, including sensation, motor skills, and cognition. Provided by the Human Connectome Project, we make use of three cohorts: 640 participants for model training, 160 subjects for validation, and 200 subjects for model performance testing thus enhancing prediction generalization. Our modeling consists of two main stages, namely dimensionality reduction in brain network features at multiple scales, followed by canonical correlation analysis, which determines an optimal linear combination of connectivity features to predict multiple behavioral performance scores. To assess the differences in the predictive power of each modality, we separately applied three different strategies: structural unimodal, functional unimodal, and multimodal, that is, structural in combination with functional features of the brain network. Our results show that the multimodal association outperforms any of the unimodal analyses. Then, to answer which human brain structures were most involved in predicting multiple behavioral scores, we simulated different synthetic scenarios in which in each case we completely deleted a brain structure or a complete resting state network, and recalculated performance in its absence. In deletions, we found critical structures to affect performance when predicting single behavioral domains, but this occurred in a lesser manner for prediction of multi-domain behavior. Overall, our results confirm that although there are synergistic contributions between brain structure and function that enhance behavioral prediction, brain networks may also be mutually redundant in predicting multidomain behavior, such that even after deletion of a structure, the connectivity of the others can compensate for its lack in predicting behavior.

## 1. Introduction

Simultaneous mapping of multiple behavioral domains onto brain networks is a major challenge for modern neuroscience. While it is true that the neuroscience community often assumes that different behavioral domains are encoded in distinct brain networks, the precise mapping between multidomain behavior and brain networks is largely unknown. Here, we use brain images and neurobehavioral scores from *N* = 1,000 healthy participants and perform state-of-the-art machine learning analyses combining structural and functional brain networks at different spatial resolutions with comprehensive behavioral assessments within the domains of sensation, motor skills, and cognition. Previous studies addressed correlative relationships between brain network connectivity and neurobehavioral measures, such as motor function (Raichlen et al., 2016; Lo et al., 2017; Boyne et al., 2018), cognitive tasks (Zimmermann et al., 2018; Yu et al., 2020; Rasero et al., 2021), and sensory experiences (Yeung et al., 2016; Spisak et al., 2020; Park et al., 2020). Some relevant questions arise from these studies: Which modality is the one that dominates the association across behavioral domains? In other words, is the association performance higher when we use features extracted exclusively from structural brain networks or when we do it from functional ones? Is it rather greater when combining the two structural and functional modalities? Does this answer depend in any way on which particular behavioral domain we are referring to?

Many previous studies have advanced in these directions. Some authors have used resting prediction capabilities to understand general cognition (Song et al., 2008; Moeller et al., 2015; Hearne et al., 2016; Smith, 2016; Ferguson et al., 2017), while others incorporated prediction power by adding features of structural networks (Matejko et al., 2013; Klein et al., 2016; Lin et al., 2020; Dhamala et al., 2021). In addition to the modality from which the brain network is built, the spatial scale at which network nodes interact is critical in systems neuroscience (Churchland and Sejnowski, 1992). Although synaptic connectivity networks account for interactions at the cellular scale, and therefore, they are inaccessible networks from magnetic resonance imaging, on a macroscale brain networks represent interactions between different populations (Craddock et al., 2013; Diez et al., 2015). These interactions appear to be organized hierarchically, where nodes are progressively merged together into modules following a nested hierarchy (Bassett et al., 2008, 2011; Betzel et al., 2013, 2014; Petersen and Sporns, 2015; Bassett and Sporns, 2017; Diez et al., 2017; Bonifazi et al., 2018; Ashourvan et al., 2019; Suárez et al., 2020). For such a class of hierarchical networks, one could define different levels deep in the hierarchy and build a different brain network at any fixed level. When we combine different network metrics at different scales, we refer to multiscale computing.

In the present study and motivated by previous works (Smith et al., 2015; Salvan et al., 2021; Taquet et al., 2021), we have performed a machine learning analysis based on canonical correlation analysis in combination with dimensionality reduction and cross-validation techniques to overcome overfitting, to perform brain network mapping of multiple neurobehavioral domains. The characteristics of the network are multimodal, extracted from a combination of functional and structural networks, and multiscale, using the hierarchical atlas of the brain (Diez et al., 2015), used in other previous studies (Rasero et al., 2017; Bonifazi et al., 2018; Camino-Pontes et al., 2018; beim Graben et al., 2019; He et al., 2020; Fernandez-Iriondo et al., 2021; Gatica et al., 2021).

## 2. Materials and Methods

### 2.1. Participants

In this work we have used open access data from the Human Connectome Project (HCP). In particular, raw images and neurobehavioral scores were taken from *N* = 1,000 healthy adult subjects (ages between 22 and 37 years, mean = 28.68, SD = 3.69), of whom 536 were women and 464 men.

HCP ensures confidentiality through rigorous deletion of personal data followed by alphanumeric coding of all data modalities, so that the patient's name or personal data will not appear in any publication or communication from these results.

Participants were divided into three cohorts: 640 participants for model training, 200 subjects for model performance testing, and 160 subjects for validation.

### 2.2. Neuro-Behavioral Measurements

Behavioral scores resulted from the NIH Toolbox for Assessment of Neurological and Behavioral Function^1^, a set of brief psychometrically sound measures to assess motor, emotional, sensory, and cognitive function valid in people aged 3–85. In particular for this study, we made use of tests scores from the domains of sensation, cognition, and motor skills. Table 1 summarizes the different domains, the name of the tests and the score statistics for each test.

**Table 1:** Brief description of neuro-behavioral measures from the NIH Toolbox.

**Domain**	**Subdomain**	**Measurement**	**Raw value (μ±σ)**
Cognition	Attention executive functioning	Flanker task	111.92, 10.04
	Language	Picture vocabulary	117.31, 9.49
	Processing speed	Pattern completion processing speed	115.35, 15.36
	Episodic memory	Picture sequence memory	112.44, 13.21
	Executive function	Dimensional change card sort	115.34, 10.18
	Working memory	List sorting	111.58, 11.15
	Language	Oral reading recognition	117.27, 10.45
Motor	Dexterity	9-hole Pegboard	112.67, 10.72
	Locomotion	4 m walk test	1.31, 0.19
	Endurance	2 min walk test	110.61, 11.87
Sensation	Olfaction	Odor identification test	110.66, 9.02
	Pain	Pain intensity and interference surveys	1.35, 1.67
	Taste	Taste intensity test	94.88, 14.36
	Audition	Words in noise	4.32, 1.48

In total, there were 23 missing values in the neurobehavioral scores. In the cognitive domain, 1 participant had no value in *Picture Sequence Memory* and two participants in *Dimensional Shift Card Sorting*. In the sensory domain, two subjects lacked scores in *Smell Identification*, four participants in *Pain Intensity*, five participants in *Taste Intensity*, and eight participants in *Words in Noise*. Finally, in the motor domain, only one participant had a missing value on the *2 min walk test*. All these missing values were imputed using a k-nearest neighbors strategy, which is a distance-based multivariate imputation strategy that has shown good convergence between observed and imputed data (Llera et al., 2022).

### 2.3. Image Acquisition

For each HCP subject, MRI acquisition was performed using a 3T Siemens Connectome Skyra with a 100 mT/m and 32-channel receive coils.

#### 2.3.1. Anatomical Data

High-resolution T1-weighted images were acquired with a 3D magnetization prepared rapid acquisition gradient echo (MPRAGE) and the following scanning parameters: repetition time (TR) = 2, 400 ms, echo time (TE) = 2.14 ms, voxel size = 0.7 × 0.7 × 0.7 mm^3^, slice thickness = 5.0 mm, flip-angle = 8 deg, field of view (FOV) = 224 × 224mm^2^ and acquisition-time = 7 min and 40 s.

#### 2.3.2. Resting State Functional Data

An EPI sequence was applied with a duration of 14 min 33 s and the following parameters: 1,200 brain volumes, TR = 720 ms, TE = 33.1 ms, FOV = 208 × 180 mm^2^, flip-angle = 52 deg, voxel size = 2 × 2 × 2 mm^3^, matrix = 104 × 90, slice thickness = 2.0 mm, and 72 slices per volume.

#### 2.3.3. Diffusion Data

An EPI diffusion sequence was applied with the following parameters: TR = 5,520 ms, TE = 89.5 ms, voxel size = 1.25 × 1.25 × 1.25 mm^3^, slice thickness = 1.25 mm, FOV = 210 × 180 mm^2^, 111 slices per volume, matrix = 168 × 144, flip-angle = 78 deg, 90 diffusion weighting directions (*b* ≠ 0), and six *b* = 0 acquisitions, three shells of b = 1,000, 2,000 and 3,000 s/mm^2^ and acquisition time = 9 min 50 s.

For more details on MRI acquisition parameters, please refer to the documentation on the HCP official website^2^.

### 2.4. Image Preprocessing

#### 2.4.1. Functional Images

Resting-state functional magnetic resonance imaging of *N* = 1,000 healthy HCP controls were used for this study. First, the images were corrected for gradient distortions and normalized to the standard MNI152 template of voxel size equal to 2 × 2 × 2 mm^3^ using the HCP pipelines *fMRIVolume* and *fMRISurface*. After image normalization, we eliminated nuisances with a procedure that combines a volume censoring strategy and motion-related time course regression, along with physiological signal regression. For this, the volumes were marked as censored when the frame displacement (FD) was greater than 0.2 or the root mean square derivative of the variance was >0.75%, following previous recommendations (Power et al., 2013, 2014; Parkes et al., 2018). In addition, the volume before the censored one and the two after it were also marked as censored. Next, the entire time series was divided into segments of five volumes in length, to finally eliminate all the segments that contained at least one contaminated volume, as well as the first segment. After that, nuisances were removed while simultaneously applying a bandpass filter between 0.01 and 0.08 Hz. Nuisance signals were the first five principal components of the CSF and WM signals; linear and quadratic trends; and the 24-parameter motion-related time series. Finally, each filtered image was spatially smoothed with a 6 mm FWHM Gaussian kernel.

#### 2.4.2. Diffusion Images

We first made use of bedpostx (Jbabdi et al., 2012) images obtained after applying the HCP pipeline and used Camino software (Cook et al., 2005) to perform deterministic tractography with fiber assignment using the continuous tracking algorithm (Mori et al., 1999), using a maximum curvature of 60° and a fractional anisotropy threshold of 0.15.

### 2.5. Brain Partition for Multimodal and Multi-Scale Networks

First, we identified the 50 participants with the lowest number of motion-censored frames in the functional sequence (the number of 50 was chosen simply to reduce computational cost and maintain a sufficient number of participants to define population matrices). Next, and similar to Diez et al. (2015), we performed an unsupervised clustering voxel-level functional data to define a large number of microregions, following Craddock et al. (2012), which will define the highest spatial resolution scale used for subsequent analyses. In contrast to Diez et al. (2015), where all the voxels were used for clustering, here we applied eight distinct clustering calculations, in each one we only considered the voxels contained in the following macroregions: frontal lobe, parietal lobe, occipital pole, temporal pole, insula, cingulate cortex, cerebellum, and subcortical structures (pooling together the thalamus, caudate, putamen, pallidum, amygdala, hippocampus, and brainstem). In this way, although we used a functional partition, the final regions had the anatomical restrictions defined by these macroregions. After pooling all the microregions from the eight different partitions, we had a whole-brain partition of 2,308 microregions with a mean size of 76 voxels (range 20–131).

### 2.6. Calculation of SC and FC Matrices for Definition of Brain Connectivity Features

Both SC and FC were connectivity matrices of 2,308 nodes equal to the number of microregions obtained in the previous section. Each SC matrix element was obtained by counting the number of white matter streamlines connecting a given node pair, while FC elements were calculated by evaluating the pairwise Pearson correlation coefficient between node time series. To obtain connectivity features, and similar to Diez et al. (2015), we performed a hierarchical agglomerative clustering applied to the spatial concatenation of the FC and SC matrices. This approach provided a hierarchical tree or dendrogram in which nodes were progressively merged into modules following a nested “neighborhood” hierarchy. Cutting this tree at any arbitrary level leads to a combination of the initial 2,308 microregions into a finite number of modules (*M*) that can be specifically tuned by varying the depth of the cut. Although many modules appeared repeatedly along the tree, we only considered one instance of the repeated modules for the computation of the machine learning connectivity features, which we will call unique modules from here on. The combination of different scales (here obtained from matrices ranging from *M* = 20 to *M* = 1, 000 modules), provided different multiscale connectivity features. Here, following Bonifazi et al. (2018), we computed for each module at a given dendrogram level, four feature classes:

- *Functional Internal Connectivity (FIC)*: Mean absolute value of the functional weights of all links within the given module.
- *Functional External Connectivity (FEC)*: Mean absolute value of the functional weights of all the links that connect the regions within that module with other regions of the brain.
- *Structural Internal Connectivity (SIC)*: Average value of the structural weights of all links within the given module.
- *Structural External Connectivity (SEC)*: Average value of the structural weights of all the links that connect the regions within that module with other regions of the brain.

Modules with a single microregion were discarded for all analyses. Furthermore, and because each of the 2,308 microregions is constructed with the same (on average) number of voxels, and because we take the average interaction intra- and inter-module, the connectivity features that we have used in our machine learning analysis accounts for the variability that arises from differences in size of both microregions and modules.

### 2.7. Neuro-Behavioral Scores and Brain Connectivity Association Through Canonical Correlation Analysis

Principal component analysis (PCA) is a widely used statistical method to reduce the dimensionality of data. From the raw data matrix *X* ∈ ℝ^*N* × *F*^ where *N* is the number of subjects and *F* the number of features, PCA aims to project the original data in a new space *N* × *D* such that *D* ≪ *F* and where the variance of the projected data is almost equal to the variance of the original. In this way, the new projected data has a lower dimension than the original data. We first applied PCA to the connectivity feature matrix. We then applied canonical correlation analysis (CCA) to find an optimal linear combination of the previously obtained principal components (*PC* ∈ ℝ^*N*×*D*^) that maximizes the correlation with a linear combination of *Q* different behavior scores (represented in the variable *Y* ∈ ℝ^*N*×*Q*^). Thus, given the components *PC* and the behavior scores *Y*, the CCA finds for the linear combinations *U* ≡ *PC*·*A* and *V* ≡ *Y*·*B*, and where the coefficients *A* ∈ ℝ^*D*×*D*^ and *B* ∈ ℝ^*Q*×*Q*^ are obtained after maximizing the canonical correlation coefficient, defined as:


$$R=\frac{\sum \left(\vec{U1}-\bar{U_{1}}\right)\left(\vec{V_{1}}-\bar{V_{1}}\right)}{\sqrt{\sum {\left(\vec{U_{1}}-\bar{U_{1}}\right)}^{2}\sum {\left(\vec{V_{1}}-\bar{V_{1}}\right)}^{2}}}.$$

Here, $\vec{U_{1}}$ and $\vec{V_{1}}$ represent the vector representation of the first canonical mode in CCA, and $\bar{U_{1}}$ and $\bar{V_{1}}$ represent respectively the mean values of $\vec{U_{1}}$ and $\vec{V_{1}}$. The maximum value of *R* is denoted by *T*.

The statistical significance of the canonical correlation coefficient *R* was tested by constructing the null hypothesis distribution of surrogates by performing 2,000 random permutations on the Y-labels and calculating the significance (*p*-value) of the actual *R* within the surrogate distribution.

### 2.8. Machine Learning Strategy With Training, Validation, and Test Datasets

We randomly divided all participants into three groups to avoid overfitting problems and achieve a better generalization of our predictions: training set (64%), validation set (16%), and test set (20%). We first started with the training set and applied PCA to all the connectivity features and this provided a set of principal components. PCs are a linear combination of the original variables, so we applied this resulting combination to the validation set. To decide the number of PCs ultimately used for the model, also known as model order selection, we constructed two different CCA performance curves as a function of the number of PCs. The first was constructed from the PCs and neurobehavioral scores in the training dataset; the second one in the validation set, but in this case the canonical variables *U* and *V* were derived from the mixed matrices *A* and *B* learned in the training data set, respectively, *A*_*train*_ and *B*_*train*_. The number of PCs that maximized CCA performance on the validation curve defined the model order for our analysis, which was then used on the test data set. In particular, after dimensionality reduction on the test dataset by the given number of components (model order), a CCA was applied to search for the underlying associations by maximizing the correlation coefficient between *U* ≡ *PC*_*test*_·*A*_*train*_ and *V* ≡ *Y*_*test*_·*B*_*train*_.

### 2.9. Brain Maps Representation of Maximum Association Solutions

PCs from the original matrix of brain connectivity features were used as independent variables for the CCA to find the maximum association with behavioral scores. Therefore, the resulting variables of CCA can be written as the dot product:


$$W=W_{PCA}\cdot W_{CCA},$$

where $W_{PCA}\in {\mathbb{R}}^{F\times D}$ is the rotation matrix composed of columns of eigenvectors PCA and $W_{CCA}\in {\mathbb{R}}^{D\times 1}$ is the vector of coefficients corresponding to the first canonical variable (*U*_1_). Since the vector *W* has dimension *F*, each component represents the weight of each of the original features. After calculating the absolute values of *W*, brain maps were constructed by coloring the different connectivity features associated with each module.

### 2.10. Synthetic Complete Deletion of Brain Macroregions

To quantify the impact that the connectivity of each macroregion *i* had on the prediction of the behavior, we simulated synthetic deletions of macroregions and recalculated new connectivity matrices in their absence. For the first part of this study, the brain macroregions were the same eight used to construct the multiscale brain partition, namely frontal lobe, parietal lobe, occipital pole, temporal pole, insula, cingulate cortex, cerebellum, and the junction of various subcortical structures; for the second part, macroregions were defined as the number of microregions overlapping at least 50% with each of the seven resting-state networks proposed by Yeo et al., including cortex (Thomas Yeo et al., 2011), cerebellum (Buckner et al., 2011), and striatum (Choi et al., 2012). Next, we computed an index ρ as the performance ratio between each synthetic network with macroregion *i* absent and the actual network (where all macroregions were present), i.e.,


$$\rho_{i}\equiv \frac{R_{i}^{2}\;(i\text{ absent})}{R^{2}\;(\text{all present})}.$$

The index *i* represents a given deleted macroregion. Two different scenarios occurred:

- ρ_*i*_ < 1 : *Constructive role of the macroregion i*. The connectivity matrix with deleted *i* performed worse compared to when it was not deleted, therefore, the connectivity of the macroregion *i* to the rest of the brain had a constructive role in predicting the behavior.
- ρ_*i*_ ≈ 1 : *Irrelevant role of macroregion i*. The connectivity matrix with deleted *i* had almost the same performance compared to when it was not deleted, therefore the connectivity of the *i* macroregion had an irrelevant role in predicting the behavior.

## 3. Results

**Figure 1:**
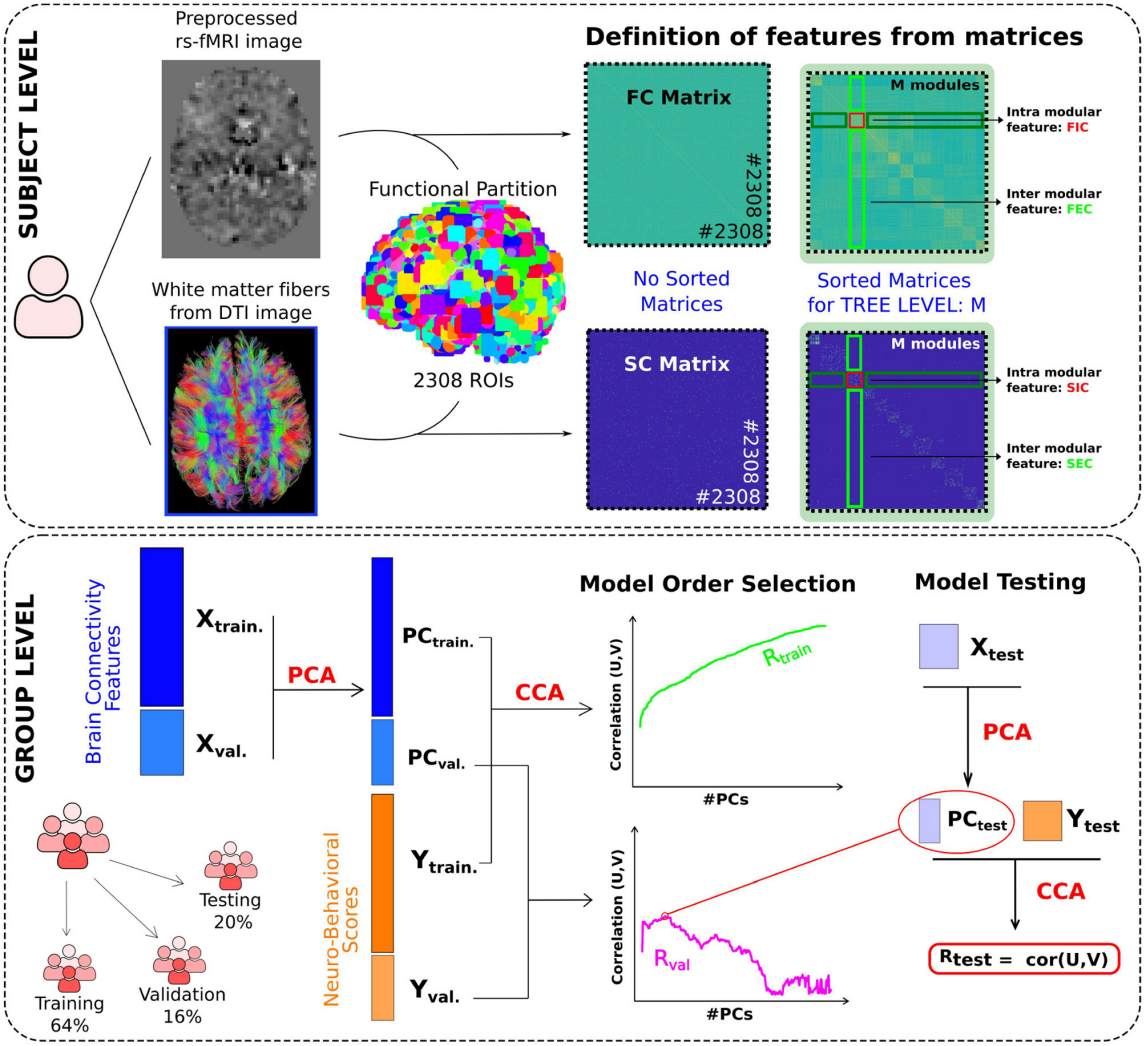
Brain mapping of neuro-behavioral scores using multimodal and multiscale networks through canonical correlation analysis. Subject level: FC and SC brain networks were built respectively from two data modalities: resting functional imaging (rs-fMRI) and diffusion tensor imaging (DTI). An initial functional brain partition of 2,308 microregions (ROIs) was built, which represents the lowest level (with highest spatial resolution) of our hierarchical partitioning. For the different M levels in the hierarchical tree (here, we varied M from 20 to 1,000), we built for each module in the M level four classes of connectivity features: FIC, FEC, SIC and SEC. This procedure provided a total number of 5,208 multi-scale connectivity features for each subject, 2,587 structural and 2,621 functional, which were used for the following analyses. Group level: Three different cohorts have been used, Training, Validation and Testing. The training and validation datasets were used for selecting the number of principal components (PCs) to reduce the original X dimensionality, containing all connectivity-features. Such a number, considered here as the model order, will be finally used to predict the neuro-behavioral scores (Y) by means of CCA in the test dataset, which provides the final performance (measured by Rtest) in the association between connectivity and behavior.

A population of healthy young participants (*N* = 1, 000) was studied and, in particular, their anatomical, diffusion and functional images were used, as well as their neurobehavioral scores (Figure 1). We first calculated the SC and FC matrices for the brain partition of 2,308 microregions. Next, we computed from the module-level connectivity matrices the features of FIC, FEC, SIC, and SEC for each of the unique modules along the hierarchical tree. In particular, using 50 different levels, from *M* = 20 to *M* = 1, 000 with a step Δ*M* = 20, this procedure yielded a total number of 5,208 multiscale connectivity features for each participant, 2,587 structural and 2,621 functional, which were used for the following analyses.

Before starting machine learning analyzes to predict behavior, we first applied the Z-score to all neurobehavioral scores and connectivity features. Using the three training, validation and test cohorts, and following the strategy depicted in Figure 1, a preliminary analysis showed that for the *strength* behavioral score the corresponding CCA coefficient was extremely high as compared to the rest (Supplementary Figure 1), so we decided to omit that score for further analyses.

**Figure 2:**
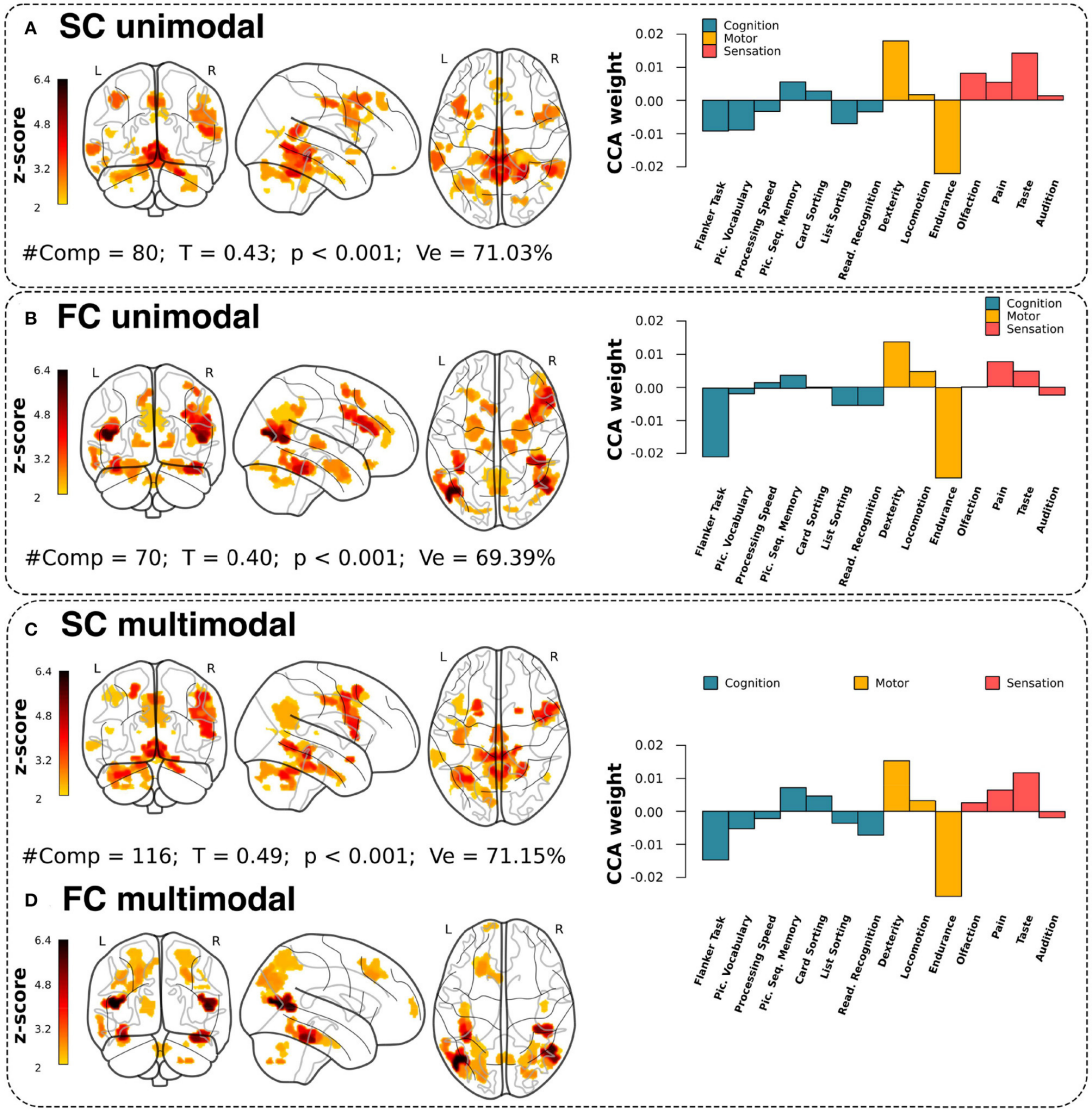
Brain maps and CCA weights for unimodal and multimodal associations. Final maps built from the multidomain CCA solution with the highest correlation coefficient (T) correlation between the X variables (the PCA components from each modality) and the Y variables (neuro-behavioral metrics from multiple domains). From top to bottom, **(A)** SC unimodal, **(B)** FC unimodal, and **(C,D)** SC+FC multimodal, plotting separately the SC and FC contributions. **(A–D)** Number of PCs used in the model (#PC), maximum correlation in the association achieved by the first canonical mode (T), p-value statistical significance (p), and the amount of variance explained after PCA (Ve). For visualization, all maps were threshold to values such *Z* > 2. The right panels provide the CCA weights for each neuro-behavioral domain at the maximum association between connectivity and behavior.

Three different strategies were applied: structural unimodal, considering only SIC and SEC features, functional unimodal (based on FIC and FEC), and multimodal, combining all feature classes. For all three strategies, we predicted multidomain behavioral performance by combining sensation, motor skills, and cognition scores. Regarding the association performance (Figure 2), the maximum canonical correlation coefficient (*T*) was higher in the multimodal analysis compared to the unimodal one, that is, *T*= 0.49 (*p* < 0.001) vs. *T*= 0.40 (functional unimodal, *p* < 0.001) or *T*= 0.43 (structural unimodal, *p* < 0.001). Furthermore, related to PCA dimensionality reduction, we were able to reduce in all three strategies the initial multiscale connectivity features of 5,208 to the order of one hundred principal components (see #comp varying between 70 and 116 in Figure 2) keeping the explained variance relatively high (Ve ranging in 69 and 71%).

Looking at which circuits were responsible for association between brain connectivity and behavior, the purely structural contributions, and thus not appearing in the functional form, were the medial orbitofrontal, superior temporal, lateral occipital, and transverse temporal. Similarly, there were purely functional contributions for the entorhinal, amygdala, hippocampus, bankssts and lateral-orbitofrontal, while the areas that participated in both structural and functional representations were the superior-parietal and the lingual.

To assess the functional characterization of these maps, we overlaid them with known resting-state networks (Thomas Yeo et al., 2011). Supplementary Table 1 shows that the unimodal structural maps had a higher overlap with the somatomotor network (with a total overlap of 4.56%), while the unimodal functional maps were better characterized by the frontoparietal network (6.88%). However, for the multimodal association (Supplementary Table 2), the structural map had a higher representation in the default mode network (4.96%), while the functional maps mostly overlapped with dorsal attention network (10.23%). For comparison purposes, Supplementary Tables 1, 2 also provide the overlap between brain maps and resting-state networks for the unidomain behavioral association along with Supplementary Figure 2 showing the domain-by-domain results with the CCA weights of each behavioral score and the associated brain circuits as maps.

Our CCA analysis was used to maximize the association between brain connectivity features and multiple behavioral domains. Looking at the CCA behavioral weights corresponding to the maximal association solution (right panel plots in Figure 2), we observe higher weights for the motor domain, and in particular for *resistance* and *skill* scores. Furthermore, it is important to note that the three solutions provided a very similar pattern in scores and domains. Therefore, although at the level of brain maps there were differences between the different strategies (structural unimodal, functional unimodal, and multimodal), the distribution of behavioral weights remained almost invariant, which shows that the connectivity characteristics of the different circuits can associate with certain invariance towards multivariate behavioral repertoires.

**Figure 3:**
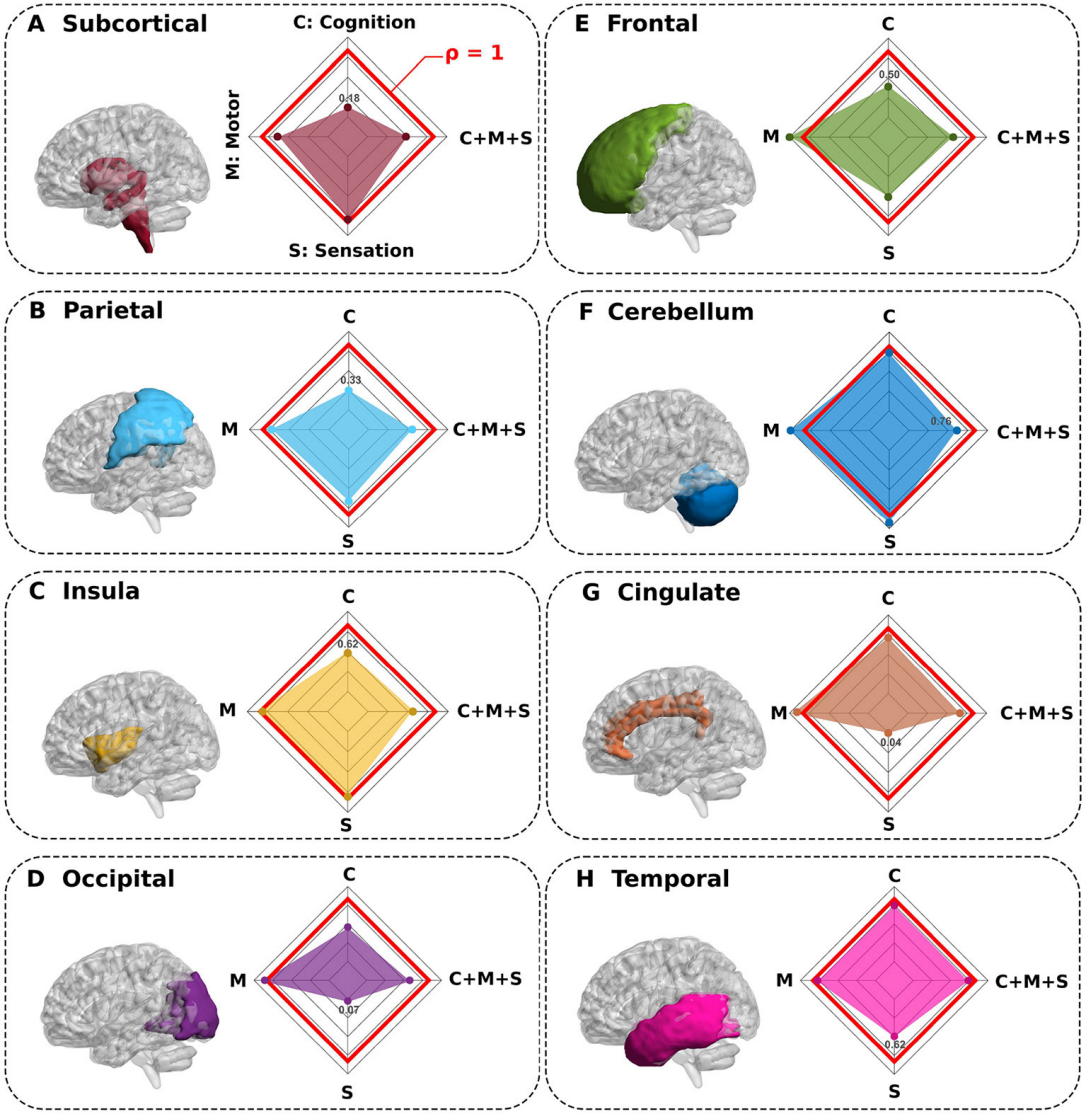
Synthetic complete deletions of brain macroregions and their impact in predicting neuro-behavioral scores. **(A–H)** Eight different macroregions were completely deleted (separately one by one) to quantify the impact that each macroregion connectivity has in predicting behavior. In particular, after a macroregion deletion, we recalculated new connectivity matrices in its absence and re-calculated the association with behavior for this novel situation. All panels show the macroregion deleted and the performance ratio ρ between the synthetic deletion as compared to the situation where all regions were present (spider plot). While values of ρ close to 1 (red line in the spider plot) show regions such that their deletions do not greatly impact the behavior prediction, values smaller than 1 show the regions with highest contribution in predicting behavior (for each spider plot, the minimum value is highlighted).

In order to answer the connectivity of which structures were most important in predicting behavioral performance, we simulated complete macroregion deletions, represented by the index *i*, and recomputed new connectivity matrices in their absence. The comparison between the synthetic complete deletion of *i* and the real performance (where all the macro-regions were present) allowed us to define the ratio ρ_*i*_ (the lower it is, the greater the contribution of the connectivity of the *i* macroregion). Indeed, this index provided very useful information –interventionist– about the contribution that *i* had in predicting behavior. Surprisingly, Figure 3 shows that by deleting the *subcortical* and *parietal* macroregions, the prediction performance of the cognitive domain was drastically reduced. Similarly, for sensation performance prediction, deletion of the *cingulate* and *occipital* regions provided the largest decrease. In contrast, for the ρ_*i*_ ≈ 1 situations, we conclude that the connectivity of those macroregions played an irrelevant role in predicting behavior, since with and without the deletion of *i* it provided equivalent performance. This indeed occurred for motor performance (M), where none of the deletions affected drastically its prediction.

We also found that although there were critical structures that played a crucial role in predicting cognition (C) and sensation (S) domains, however, those structures were no longer relevant when predicting multidomain C+M+S behavior. Therefore, for this situation, regardless of which structure we deleted, the connectivity of other regions compensated for their predicting participation with similar performance.

**Figure 4:**
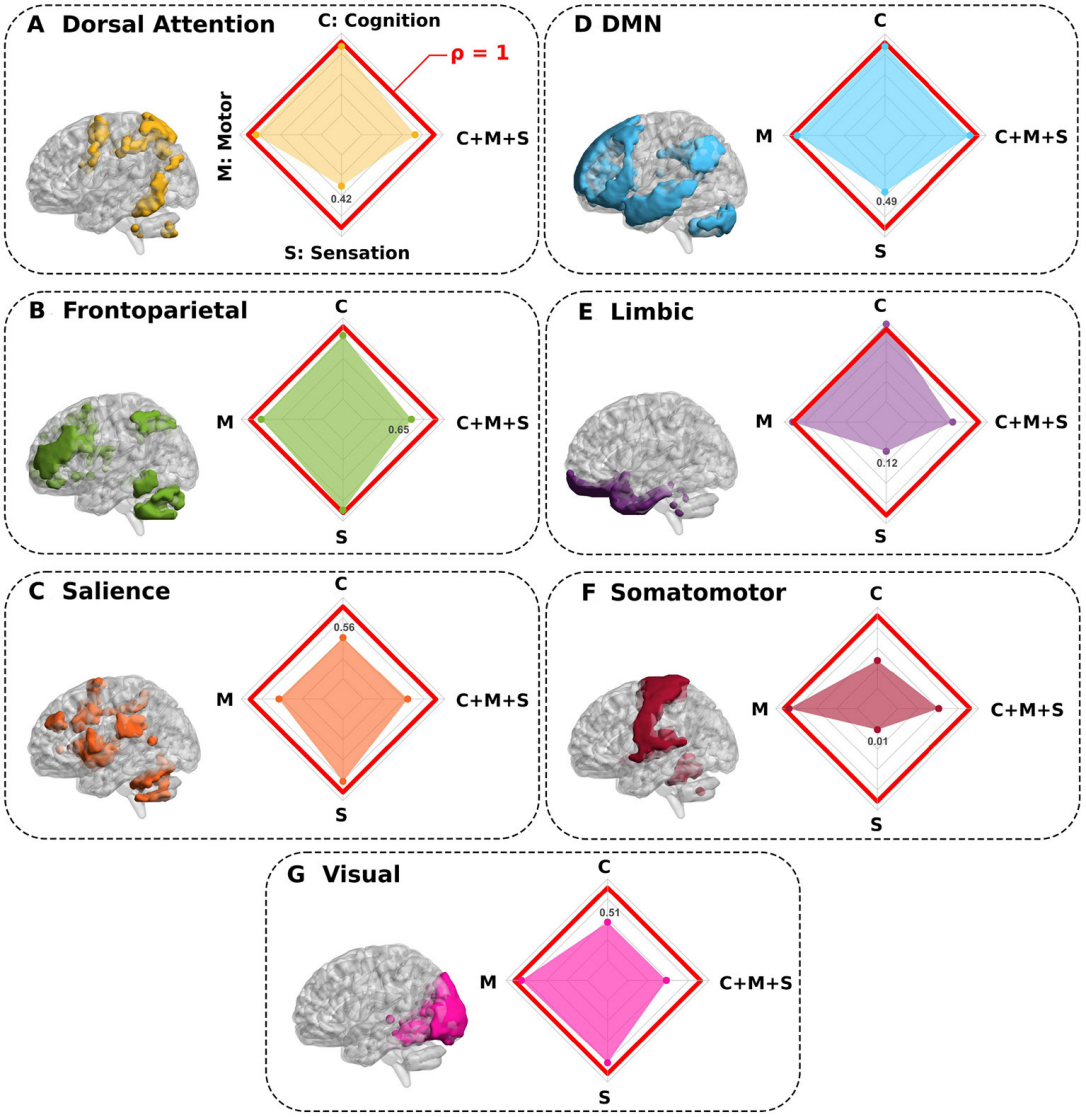
Synthetic complete deletions of functional resting state networks and their impact in predicting neuro-behavioral scores. **(A–G)** Seven different functional networks were completely deleted (separately one by one) to quantify the impact that each specific network has in predicting behavior. In particular, after a network deletion, we recalculated new connectivity matrices in its absence and re-calculated the association with behavior for this novel situation. All panels show the network deleted and the performance ratio ρ between the synthetic deletion as compared to the situation where all regions were present (spider plot). While values of ρ close to 1 (red line in the spider plot) show regions such that their deletions do not greatly impact the behavior prediction, values smaller than 1 show the networks that had the highest contribution in predicting behavior (for each spider plot, the minimum value is highlighted).

Finally, to further advance and test other scenarios, we also simulated the complete elimination of well-known resting-state networks. As a result, Figure 4 shows that by deleting the *salience, somatomotor* and *visual* networks, the prediction performance of the cognitive domain was strongly reduced. In a like manner, for sensation performance prediction, the deletion of the *dorsal attention, default mode network (DMN), limbic*, and *somatomotor* networks revealed the largest decrease. For the motor domain, the *salience* was the only network contributing to reduction in prediction performance, somehow, indicating that the motor skills encoded in our scores are not specific to any of the other networks. For the multidomain C+M+S situation, all networks (except the DMN) had an effect on prediction performance and major decreases occurred for *visual, salience*, and *somatomotor* network removal.

## 4. Discussion

Mapping behavior in brain networks across multiple discrete domains remains a major challenge. Here, we have developed a multi-scale, multi-modal, multi-domain strategy to assess this problem. Multiscale because the connectivity features were built from networks of different sizes, and multimodal because we built them from both functional and structural networks. Rather, multidomain refers to the point at which behavioral performance takes into account multiple scores that assess cognitive, sensory, and motor skills. Our modeling approach first applied dimensionality reduction on the ensemble of brain connectivity features, followed by regression-like associations using canonical correlation analysis.

Our results have shown that, in general, multimodal structure-function connectivity features outperformed the prediction achieved by any of the unimodal forms (either structural or functional), suggesting synergistic contributions between them to improve behavioral prediction. It is important to highlight that the contribution between the two modalities is systematically balanced in all the scales, since there is no specific scale where one modality predominates over the other, in a statistically significant way (Supplementary Figure 3). This fact reinforces the use of multimodal and multiscale computation to predict behavior, because the proportion of weights coming from structural networks with respect to functional ones is balanced, and all scales are needed as there are no fixed dominant ones.

In the first part of the study, our analysis based on synthetic deletions of anatomical macroregions have shown that the connectivity of some specific brain structures played an important role in predicting domains of cognition and sensation separately, but this was not the case for the motor domain. Thus, we found that connectivity to the parietal lobe made a key contribution to predicting cognition, in agreement with previous research highlighting parietal localization while performing cognition tasks (Culham and Kanwisher, 2001). Indeed, the parietal lobe has been shown to mediate different connectivity projections, reflecting its role as a central hub for perception, action, and cognition (Gottlieb, 2007). Furthermore, also for the prediction of cognition, our results showed the relevance of subcortical-cortical connections in agreement with previous work (Münte et al., 2008; Zonneveld et al., 2019). Of note, the authors in Riveros et al. (2019) also reported the importance of the fronto-subcortical pathway for cognitive abilities, which is also in agreement with our results showing that after deletion of the frontal lobe, the connectivity performance for predicting cognition was also strongly reduced. Remarkably, we also found that for predicting sensation behavior, removal of the cingulum and occipital lobe had a large impact on performance. The role of the anterior cingulate cortex within the salience network in relation to pain processing is well-known (Seeley, 2019) and also in age-related affective pain (Vogt, 2005; Lieberman and Eisenberger, 2015; Terrasa et al., 2021). On the role of occipital connectivity in predicting sensation performance, a recent study showed the involvement of the occipital gyrus in tasks related to those assessed in our cohort within the sensations domain (Hwang et al., 2019), but also under conditions related to pain such as migraine (Miller et al., 2016).

Of great interest is the fact that although for cognition and sensation domains separately there were structures whose connectivity critically affected performance, however, these structures were not relevant in the prediction of multidomain behavior. This fact could indicate in a certain way that the participation of specific networks can be compensated by others, thus maintaining a balance to preserve performance in the different domains. This is also consistent with another of our findings, namely that when looking at the distribution of CCA weights across behavioral scores and domains, all three strategies (structural unimodal, functional unimodal, and multimodal) provided a very similar pattern of CCA weights, indicating the possibility that different brain networks are capable to fit multivariate- and multidomain-behavioral performance.

In the second part of our study, we also removed complete resting-state networks one by one and evaluated the variation in prediction performance across domains. The deletion of DMN, limbic, dorsal attention and somatomotor networks had a large impact on sensation prediction, in our scores encompassing pain, taste, smell and hearing assessments. This is in agreement with previous studies, showing the key role that the limbic system plays in pain perception and motivational responses (Yang and Chang, 2019), that taste is encoded by changes in sensorimotor states (Di Lorenzo, 2021), that dorsal attention and sensorimotor participated in pain processing (Lee et al., 2021), and that the variations in DMN affected chronic pain conditions (Fomberstein et al., 2013; Alshelh et al., 2017). Similar to our findings, previous work identified the key role of salience network connectivity in predicting motor performance, affecting almost all voluntary motor actions, while its damage contributed significantly to the essential motor deficits after stroke (Rinne et al., 2018). For the prediction of cognition, our results showed the relevance of the salience network, in agreement with previous findings showing its role as a hub for integration of cognition and attention (Menon and Uddin, 2010; La Corte et al., 2016). We also found that somatomotor and visual networks affected cognition, in full agreement with recent work showing that the majority of the cognitive decline occurring in the aging brain was related to visual and sensorimotor age-related deterioration (Stumme et al., 2020).

Future work should combine synthetic macroregion deletions with brain mapping of other cognitive domains, in both task-fMRI or resting, to reveal the contributions of brain structures that affect performance. In addition, it is worth exploring in the presence of acquired brain damage the decrease in performance in behavioral prediction, characterizing in some way more disabling lesions at the behavioral level.

## Data Availability Statement

The original contributions presented in the study are included in the article/Supplementary Material, further inquiries can be directed to the corresponding author/s.

## Ethics Statement

The studies involving human participants were reviewed and approved by HCP Consortium Ethical Committee. All participants provided their written informed consent before recruitment in this study.

## Author Contributions

IF-I performed the analyses, made the figures, drafted the first version of the manuscript, and conceived the study. AJ-M performed the analysis and made the figures. BS and NA supervised machine learning analysis. PB supervised multi-scale brain networks methodology and conceived the study. JC supervised all research, drafted the first version of the manuscript, and conceived the study. All authors wrote the manuscript and agree in its publication.

## Funding

JC was funded by Ikerbasque: The Basque Foundation for Science and by the Department of Economic Development and Infrastructure of the Basque Country (Elkartek Program Grant KK-2021-00009). AJ-M was funded by a predoctoral contract from the Department of Education of the Basque Country Predoctoral Program PRE-2019-1-0070. IF-I was funded by a research assistant contract from the University of the Basque Country (Elkartek Program Grant KK-2021/00033). PB acknowledge financial support from Ikerbasque (The Basque Foundation for Science) and FEDER (AI-2021-039).

## Conflict of Interest

The authors declare that the research was conducted in the absence of any commercial or financial relationships that could be construed as a potential conflict of interest. The handling editor JS declared a past co-authorship with the authors JC and AJ-M.

## Publisher's Note

All claims expressed in this article are solely those of the authors and do not necessarily represent those of their affiliated organizations, or those of the publisher, the editors and the reviewers. Any product that may be evaluated in this article, or claim that may be made by its manufacturer, is not guaranteed or endorsed by the publisher.

## Footnotes

^1^NIH Toolbox available at www.nihtoolbox.org.

^2^Human Connectome Project official website: https://www.humanconnectome.org/.
