# Supplementary material for: Brain Mapping of Behavioral Domains Using Multi-Scale Networks and Canonical Correlation Analysis
Source: Front Neurosci. 2022 Jun 21;16:889725. doi: 10.3389/fnins.2022.889725 (PMC9255673; doi:10.3389/fnins.2022.889725)
Supplement: Supplementary file 1 [file Data_Sheet_1.pdf]

Supplementary Material

1 SUPPLEMENTARY TABLES AND FIGURES

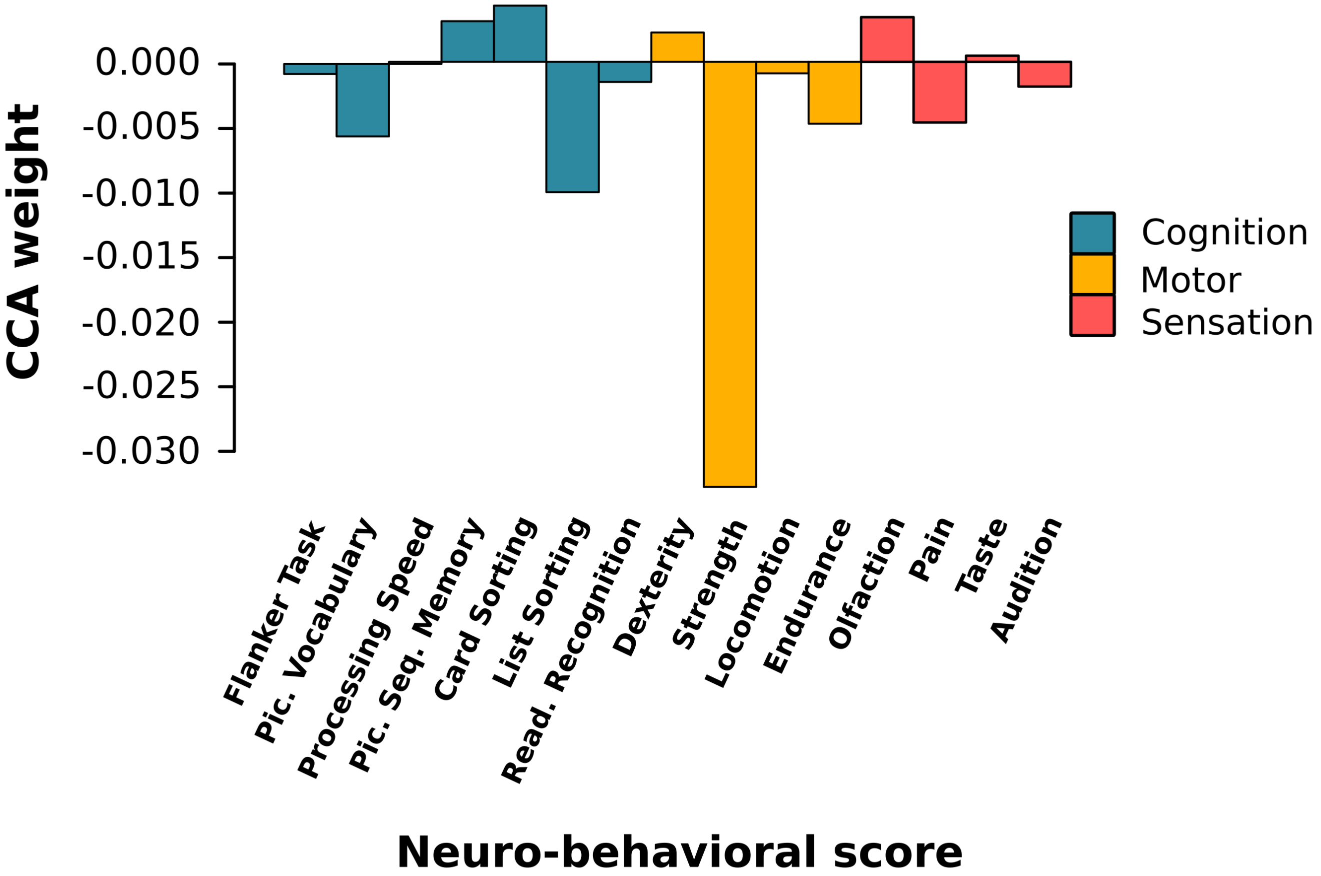

Figure S1: **CCA weights for all neuro-behavioral scores.** Because the weight for the Strength variable was much higher than others, this variable was skip for all the analysis in this work.

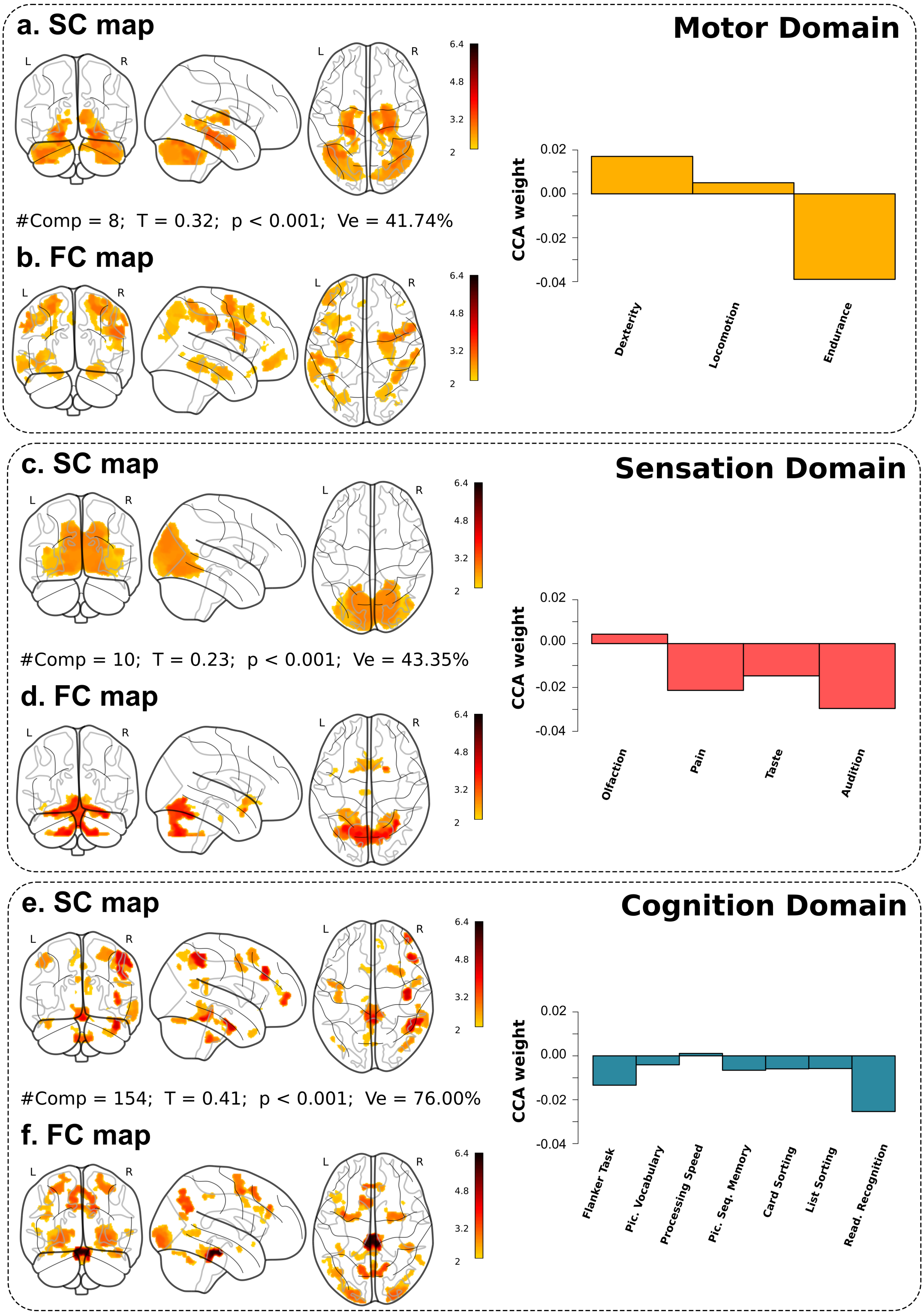

Figure S2: **Brain maps and CCA weights resulting from the association between connectivity and unidomain behavioural scores.**

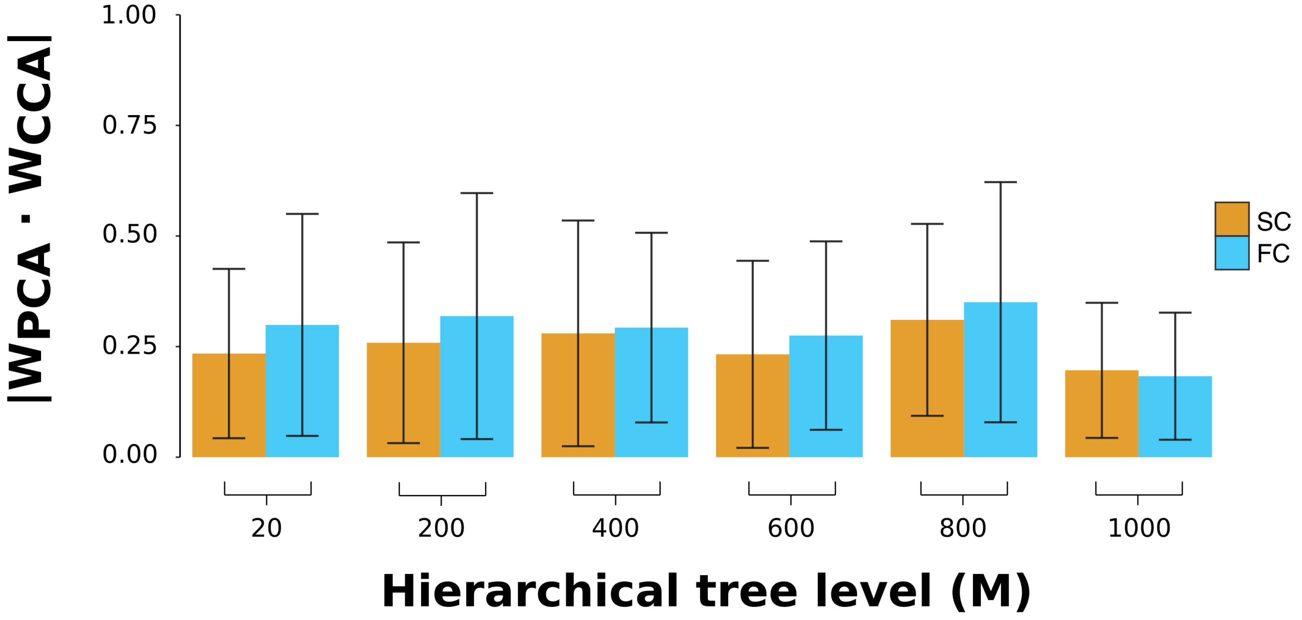

Figure S3: **Normalized total weights across multiple scales (represented by M).** Normalized total weights were built as the product between the weights of the PCA multiplied by the ones of the CCA. Histogram values across different scales, represented by the level M of the hierarchical tree. In blue we plot the histogram of functional weights and in yellow the one for structural weights.

	UNIDOMAIN						MULTIDOMAIN (C+M+S)	
	SC (%)			FC (%)				
	Cognition (C)	Motor (M)	Sensation (S)	Cognition	Motor	Sensation	SC (%)	FC (%)
**Visual**	0.99	1.06	15.91	4.25	1.33	11.15	1.00	2.89
**Somatomotor**	3.56	3.43	6.59	0.99	0.28	0.02	4.56	0.81
**Dorsal Attention**	5.08	2.69	0.86	1.63	6.54	3.30	2.87	3.58
**Salience Ventral Attention**	1.93	7.20	5.75	0.86	1.54	5.31	4.03	1.25
**Limbic**	6.16	3.34	1.29	2.54	0.33	0.67	3.65	3.30
**Frontoparietal**	3.02	7.84	0.38	1.87	3.37	3.27	4.49	6.88
**DMN**	2.28	2.29	0.06	0.98	3.87	0.47	2.90	4.62

Table S1: **Jaccard similarity index as a measure for overlapping percentage (%) between resting-state networks and resulting brain maps from unimodal analyses.** For easier reading, we colored in red the overlapping values commented in the text. DMN: Default Mode Network.

	UNIDOMAIN						MULTIDOMAIN (C+M+S)	
	SC (%)			FC (%)				
	Cognition (C)	Motor (M)	Sensation (S)	Cognition	Motor	Sensation	SC (%)	FC (%)
**Visual**	0.28	2.77	52.70	7.68	1.36	0.71	0.78	5.27
**Somatomotor**	1.38	0.12	0.08	1.11	0.95	1.78	3.71	0.29
**DorsalAttention**	3.38	0.54	0.41	4.89	14.25	2.85	3.99	10.23
**Salience Ventral Attention**	1.04	3.86	0.09	1.52	1.44	7.14	3.61	0.75
**Limbic**	3.43	1.31	0.23	1.11	1.39	3.22	3.26	2.49
**Frontoparietal**	6.20	10.42	0.02	3.81	4.45	1.43	4.29	1.94
**DMN**	1.73	8.04	0.03	1.19	5.15	1.01	4.96	2.84

Table S2: Jaccard similarity index as a measure for overlapping percentage (%) between resting-state networks and resulting brain maps from the **multimodal analysis**. For easier reading, we colored in red the overlapping values commented in the text. DMN: Default Mode Network.
